# Associations of Serum Zinc and Iron with Systemic Inflammatory Indices in Pediatric Obesity: An Exploratory Cross-Sectional Study

**DOI:** 10.3390/children13060800

**Published:** 2026-06-10

**Authors:** Mehmet Cengiz

**Affiliations:** Department of Pediatrics, Eskişehir Özel Gürlife Hospital, 26080 Eskişehir, Turkey; dr_mcengiz@hotmail.com

**Keywords:** pediatric obesity, micronutrients, zinc, iron, systemic immune-inflammation index (SII), neutrophil-to-lymphocyte ratio (NLR), inflammation, exploratory study, cross-sectional study

## Abstract

**Highlights:**

**What are the main findings?**

- Children with obesity show significantly elevated NLR, PLR, SII, and MLR compared to children without obesity.
- Iron is independently associated with inflammatory indices in multivariable regression across the full cohort.
- Zinc showed the strongest inverse correlations with inflammatory indices in the subgroup with obesity only—a hypothesis-generating observation requiring replication in larger studies.

**What are the implications of the main findings?**

- These exploratory findings support consideration of iron status assessment in pediatric obesity and warrant further prospective investigation of zinc-related inflammatory associations.

**Abstract:**

Background/Objectives: Childhood obesity is associated with chronic low-grade systemic inflammation. This exploratory cross-sectional study aimed to evaluate associations between a comprehensive panel of serum micronutrient levels and four systemic inflammatory indices—neutrophil-to-lymphocyte ratio (NLR), platelet-to-lymphocyte ratio (PLR), systemic immune-inflammation index (SII), and monocyte-to-lymphocyte ratio (MLR)—in a pediatric cohort, with iron as the primary focus and zinc as a secondary exploratory analysis. Methods: We included 410 children (mean age 7.2 ± 3.8 years; 205 male) attending a tertiary pediatric clinic. Of these, 399 had complete BMI percentile data and were included in obesity-stratified analyses; patients were classified as having obesity (BMI ≥ 95th percentile; *n* = 56) or not having obesity (*n* = 343). The remaining 11 children lacked BMI percentile data and were included only in full-cohort analyses. Serum iron, ferritin, folate, zinc, vitamin D, vitamin B12, magnesium, and phosphorus were measured alongside complete blood count parameters. Spearman correlations and multivariable ordinary least squares regression were performed. Benjamini–Hochberg false discovery rate (FDR)-adjusted q-values were computed for all Spearman correlations; all remaining analyses are exploratory and all findings should be interpreted with caution. Results: All four inflammatory indices were significantly higher in children with obesity (SII: 487.1 vs. 332.1, *p* < 0.001; NLR: 1.30 vs. 1.03, *p* = 0.001). Low serum iron was more prevalent in the group with obesity (42.9% vs. 27.1%, *p* = 0.025). In multivariable regression, serum iron was significantly associated with NLR (β = −0.009, 95% CI [−0.012, −0.005], *p* < 0.001) and SII (β = −3.268, 95% CI [−4.404, −2.132], *p* < 0.001) after adjustment for age and BMI percentile. In an exploratory analysis restricted to children with obesity and complete data (*n* = 39), zinc was associated with SII (β = −11.912, 95% CI [−21.836, −1.988], *p* = 0.025); however, the overall model was non-significant (*p* = 0.067), zinc showed no association in the full cohort, and—given the small sample and absence of multiple comparison correction—this finding must be considered strictly hypothesis-generating. Conclusions: Systemic inflammatory burden is elevated in children with obesity. Iron shows consistent associations with inflammatory indices independent of age and BMI. Zinc shows a potentially relevant, exploratory association with inflammation, specifically in the subgroup with obesity, warranting replication in adequately powered prospective studies. These findings are consistent with a role for iron status assessment in the clinical evaluation of pediatric obesity and warrant further prospective investigation of zinc-related inflammatory associations.

## 1. Introduction

Childhood obesity has emerged as one of the most critical public health challenges of the 21st century, with global prevalence continuing to rise across all age groups and socioeconomic strata [1]. Beyond its metabolic consequences—including insulin resistance, dyslipidemia, and non-alcoholic fatty liver disease—obesity in childhood is characterized by a state of chronic, low-grade systemic inflammation driven by dysfunctional adipose tissue [2,3]. This inflammatory milieu, mediated by adipokine secretion and immune cell infiltration within expanded adipose depots, is increasingly recognized as an independent contributor to long-term cardiovascular and metabolic morbidity [4].

Systemic inflammatory indices derived from routine complete blood count (CBC) parameters have attracted growing attention as cost-effective and readily available biomarkers of inflammatory burden. The neutrophil-to-lymphocyte ratio (NLR), platelet-to-lymphocyte ratio (PLR), systemic immune-inflammation index (SII = platelet × neutrophil/lymphocyte count), and monocyte-to-lymphocyte ratio (MLR) have been validated in adult populations as markers of systemic inflammation in a range of cardiometabolic and inflammatory disorders [5,6]. Data in pediatric obesity, however, remain limited.

Paradoxically, children with obesity exhibit disproportionately high rates of micronutrient deficiency despite excessive caloric intake—a phenomenon termed “hidden hunger” [7]. Iron, zinc, folate, and vitamin D insufficiencies have each been documented in pediatric obesity, attributable to poor dietary quality, increased metabolic demand, and the sequestration of fat-soluble micronutrients within expanded adipose tissue [8,9]. These micronutrients exert immunomodulatory effects: iron supports leukocyte oxidative function; zinc modulates T-cell differentiation and NF-κB-mediated inflammatory signaling; folate participates in one-carbon metabolism critical for rapidly dividing immune cells; and vitamin D acts through its receptor expressed on virtually all immune cell types [10,11,12,13].

Despite this biological plausibility, the relationship between the breadth of micronutrient status and composite systemic inflammatory indices in pediatric obesity has not been systematically evaluated. Most prior studies examine single micronutrients in isolation and rely exclusively on bivariate correlations without multivariable adjustment. The present study is primarily designed to examine the association between serum iron status and systemic inflammatory indices in a pediatric cohort, with secondary exploratory analyses evaluating the broader micronutrient panel—including zinc, folate, and vitamin D—and their relationship to inflammatory burden. Specifically, the study aimed to (1) characterize systemic inflammatory indices in children with obesity versus children without obesity; (2) compare a comprehensive micronutrient panel across BMI categories; (3) examine Spearman correlations between micronutrients and inflammatory indices in the full cohort and within the subgroup with obesity; and (4) assess multivariable associations between key micronutrients and inflammatory indices. All secondary analyses should be considered exploratory and hypothesis-generating.

## 2. Materials and Methods

### 2.1. Study Design and Participants

This cross-sectional study was conducted at the Pediatric Outpatient Clinic of Eskişehir Özel Gürlife Hospital. The study was approved by the Eskişehir Şehir Hastanesi Scientific Research Ethics Committee (Decision No: ESH/BAEK 2025/288; 11 December 2025) and conducted in accordance with the Declaration of Helsinki. This study was conducted as a retrospective review of routinely collected clinical data; individual informed consent was waived by the Eskişehir Şehir Hastanesi Scientific Research Ethics Committee, as the study involved no additional procedures or patient contact beyond standard care.

Children aged 1–18 years presenting for routine pediatric assessment were considered for inclusion. Exclusion criteria were (1) active infectious disease at the time of blood sampling; (2) known chronic inflammatory condition; (3) malignancy; (4) immunosuppressive therapy, systemic corticosteroids, iron, or micronutrient supplementation within three months prior to sampling; (5) hemoglobinopathy; and (6) incomplete laboratory data for primary study variables. After exclusions, 410 children were included in the final analysis.

### 2.2. Anthropometric Measurements and BMI Classification

Height was measured to the nearest 0.1 cm using a wall-mounted stadiometer and weight to the nearest 0.1 kg using a calibrated digital scale. BMI was calculated as weight (kg)/height^2^ (m^2^). Age- and sex-specific BMI percentiles were assigned using Turkish national reference standards. Obesity was defined as BMI ≥ 95th percentile (*n* = 56); all remaining participants with available BMI percentile data constituted the reference group without obesity (*n* = 343). Eleven children lacked BMI percentile data and were excluded from obesity-stratified group comparisons but included in full-cohort analyses. In multivariable analyses, BMI percentile was also used as a continuous covariate. The age distribution of the full cohort was as follows: preschool age (1–5 years), 165 children (40.2%); school age (6–11 years), 185 children (45.1%); adolescent (12–18 years), 60 children (14.6%). Inflammatory and micronutrient profiles vary substantially across developmental stages; accordingly, age was included as a covariate in all multivariable models.

### 2.3. Laboratory Measurements

Venous blood was collected in the morning after a minimum 8-h overnight fast. Complete blood count (CBC) with five-part differential was performed on a Sysmex XN-series automated hematology analyzer. To minimize the influence of hemolysis on zinc measurements—which are highly susceptible to spurious elevation due to erythrocyte zinc release—samples with biochemical evidence of hemolysis (elevated lactate dehydrogenase [LDH] or serum potassium above the upper limit of the reference range) were excluded from the zinc analysis. Seasonality of blood collection was not systematically recorded; this represents a limitation particularly relevant to the interpretation of vitamin D values. Biochemical parameters—including serum iron, total iron-binding capacity (TIBC), ferritin, zinc, magnesium, phosphorus, vitamin B12, folate, and 25-hydroxyvitamin D [25(OH)D]—were measured on Beckman Coulter AU680 (biochemistry) and Abbott Architect i2000SR (immunoassay) platforms using manufacturer-validated methods.

Inflammatory indices were computed as follows: NLR = absolute neutrophil count/absolute lymphocyte count; PLR = platelet count/absolute lymphocyte count; SII = (platelet count × absolute neutrophil count)/absolute lymphocyte count; MLR = absolute monocyte count/absolute lymphocyte count. Iron deficiency was defined as serum iron < 60 µg/dL. Vitamin D insufficiency was defined as 25(OH)D < 20 ng/mL.

### 2.4. Statistical Analysis

Statistical analyses were performed using Python (version 3.11; NumPy 1.25, SciPy 1.11, pandas 2.0). Normality was assessed using the Shapiro–Wilk test. Non-normally distributed continuous variables are reported as median (interquartile range, IQR); normally distributed variables as mean ± standard deviation (SD). Categorical variables are expressed as frequencies and percentages. Between-group comparisons used the Mann–Whitney U test (continuous variables) and chi-square test (categorical variables). Several potentially important confounders—including dietary intake, pubertal status, socioeconomic status, and physical activity level—were not available in the dataset and could therefore not be included as covariates; this represents a substantive limitation given that each of these variables independently influences both micronutrient levels and systemic inflammatory markers in children.

Spearman rank correlations were computed between each micronutrient and each inflammatory index across the full cohort and separately within the subgroup with obesity. Given that multiple correlations were tested across 7 micronutrients and 3 inflammatory indices, there is an elevated risk of false-positive findings. To address this, Benjamini–Hochberg false discovery rate (FDR)-adjusted q-values were computed across all 21 micronutrient–index pairs in the full cohort; results are presented alongside nominal *p*-values. No formal correction for multiple comparisons was applied to subgroup correlations or regression analyses, given the exploratory nature of those analyses; all such findings should be considered hypothesis-generating and interpreted with particular caution.

SII and NLR were selected as primary dependent variables because SII integrates platelet, neutrophil, and lymphocyte counts into a single composite index that has been validated across multiple inflammatory conditions in adults and is increasingly studied in pediatric populations, while NLR is the most widely reported and methodologically established CBC-derived inflammatory index. Multivariable ordinary least squares (OLS) linear regression was performed with SII and NLR as dependent variables and iron, zinc, folate, age, and BMI percentile as independent variables in the full cohort. Iron was prespecified as the primary predictor of interest; zinc and folate were included as exploratory secondary predictors. Prior to modelling, OLS regression assumptions were evaluated. Residual normality was assessed using the Shapiro–Wilk test; SII and NLR residuals exhibited significant right-skew (Shapiro–Wilk *p* < 0.001 for both), consistent with the known skewed distributions of these indices. Log-transformation of both outcomes substantially improved residual normality (log-SII Shapiro–Wilk *p* = 0.006; log-NLR *p* < 0.001) and yielded higher model R^2^ values (log-SII R^2^ = 0.213; log-NLR R^2^ = 0.284) compared to the untransformed models; log-transformed results are reported in Supplementary Table S1. Potential multicollinearity was assessed by computing variance inflation factors (VIF) for all predictors. VIF values were as follows: serum iron = 1.05, age = 1.22, zinc = 1.03, folate = 1.17, and BMI percentile = 1.09. All VIF values were well below the conventional threshold of 5, indicating no meaningful multicollinearity among predictors. Pairwise Pearson correlations between predictors confirmed this, with the largest absolute correlation being r = −0.367 between age and folate (Table S2). Sex was not included as a covariate in the full-cohort regression models because the sample was exactly balanced by sex (*n* = 205 male, 205 female), resulting in exact distributional balance between sexes; therefore, sex adjustment was not expected to materially alter the model estimates in this dataset. Nonetheless, known sex-related differences in iron metabolism and inflammatory marker levels mean that residual sex-related confounding cannot be fully excluded; future studies with unbalanced or larger samples should perform sex-stratified analyses. A secondary exploratory regression model was fitted within the subgroup with obesity (SII as dependent variable; zinc, iron, folate, age, sex, and BMI percentile as predictors). All tests were two-tailed; statistical significance was set at *p* < 0.05. The study was not powered for subgroup analyses and all secondary findings are exploratory.

## 3. Results

### 3.1. Demographic and Hematological Characteristics

A total of 410 children (mean age 7.2 ± 3.8 years; 205 male, 205 female) were included. The group with obesity (*n* = 56) had a median BMI percentile of 98.0 (IQR 97.0–99.0). Sex distribution was exactly balanced (28 male/28 female per group). Age was slightly but not significantly higher in the group with obesity (8.1 ± 3.6 vs. 7.0 ± 3.8 years, *p* = 0.064). Neutrophil count and platelet count were both significantly higher in children with obesity, while lymphocyte count did not differ. Detailed characteristics are presented in Table 1.

### 3.2. Micronutrient Levels by Obesity Status

Serum micronutrient concentrations are summarized in Table 2. Children with obesity had significantly lower serum iron (64 vs. 79 µg/dL, *p* = 0.003) and lower folate (9.2 vs. 10.8 ng/mL, *p* = 0.004) than peers without obesity. Ferritin was significantly higher in the group with obesity (30 vs. 22 ng/mL, *p* = 0.006), consistent with its role as an acute-phase reactant. Phosphorus was modestly higher in children with obesity (4.9 vs. 4.7 mg/dL, *p* = 0.036). Zinc, vitamin D, vitamin B12, and magnesium did not differ significantly between groups.

Low serum iron (<60 µg/dL), used here as a surrogate indicator of iron deficiency in the absence of full diagnostic work-up, was present in 24 of 56 (42.9%) children with obesity versus 93 of 343 (27.1%) children without obesity (chi-square *p* = 0.025). It should be noted that definitive diagnosis of iron deficiency requires additional markers, including ferritin and transferrin saturation; furthermore, since CRP and hepcidin were not measured—as these are not part of the routine blood panel obtained in the outpatient setting from which this dataset was derived—true iron deficiency cannot be distinguished from functional iron deficiency due to the acute-phase response. Vitamin D insufficiency (<20 ng/mL) was observed in 37.7% of children with obesity versus 35.6% of children without obesity (*p* = 0.885).

### 3.3. Systemic Inflammatory Indices Are Elevated in Obese Children

All four inflammatory indices were significantly higher in children with obesity compared to children without obesity (Table 3). SII showed the largest absolute difference (487.1 [316.0–715.9] vs. 332.1 [212.2–489.9], *p* < 0.001), corresponding to a 46.7% higher median SII in the group with obesity. MLR, NLR, and PLR also showed significant elevations. Median-stratified analyses further illustrated these patterns: children with serum iron below the cohort median (77.5 µg/dL) had significantly higher SII (424.9 vs. 305.5, *p* < 0.001); children with folate below the cohort median (10.7 ng/mL) had significantly higher NLR (1.22 vs. 0.91, *p* < 0.001); these stratified comparisons should be interpreted cautiously given the exploratory nature of all secondary analyses.

### 3.4. Spearman Correlations Between Micronutrients and Inflammatory Indices

Across the full cohort, iron showed the most consistent and statistically significant inverse correlations with inflammatory indices (Table 4). Folate and ferritin also showed significant associations. Zinc, vitamin D, vitamin B12, and magnesium showed weaker or non-significant associations in the full cohort. To address the risk of false-positive findings from multiple comparisons, Benjamini–Hochberg false discovery rate (FDR)-adjusted q-values were computed across all 21 micronutrient–index pairs and are displayed in Table 4. Iron associations with NLR, SII, and MLR remained highly significant after FDR correction (all q < 0.0001); folate associations likewise remained significant (q < 0.001 for all three indices); ferritin associations remained significant across all three indices (q < 0.001 for NLR and MLR; q = 0.005 for SII). Magnesium showed a borderline significant association with NLR that remained significant after FDR correction (*p* = 0.005, q = 0.009); however, its associations with SII and MLR did not survive correction. Zinc showed no significant association with any inflammatory index either before or after FDR correction in the full cohort (all q > 0.22).

### 3.5. Exploratory Subgroup Analysis: Zinc and Inflammatory Indices in Obese Children

Within the subgroup with obesity (*n* = 56; zinc data available in *n* = 40), zinc showed inverse correlations with inflammatory indices that were substantially stronger than those observed in the full cohort (Table 5). The Spearman correlation of zinc with SII was r = −0.555 (*p* < 0.001) and with NLR was r = −0.523 (*p* = 0.001). These correlations were also stronger than those of iron (SII: r = −0.339, *p* = 0.011) or folate (NLR: r = −0.321, *p* = 0.026) in this subgroup. These are exploratory findings derived from a small sample (*n* = 40 for zinc), and the possibility of type I error inflated by multiple testing cannot be excluded. Replication in adequately powered studies is necessary before any conclusions can be drawn.

### 3.6. Multivariable Linear Regression

Multivariable OLS regression results are presented in Table 6. In the full-cohort models (Panels A and B), serum iron was the only prespecified predictor that reached statistical significance after adjustment for age and BMI percentile. Each 1 µg/dL decrease in serum iron was associated with a 3.3-unit increase in SII and a 0.009-unit increase in NLR. To contextualize this effect: the interquartile range of serum iron in the full cohort was approximately 47 µg/dL (59–106 µg/dL); thus, moving from the 25th to 75th percentile of serum iron would be associated with an estimated 155-unit decrease in SII (≈3.268 × 47), equivalent to approximately 44% of the observed obese–non-obese SII difference. Age was also independently associated with higher inflammatory indices. Zinc, folate, and BMI percentile did not reach significance in the full-cohort models. Because residuals from the original-scale SII and NLR models were markedly non-normal (Shapiro–Wilk *p* < 0.001), log-transformed models were also fitted and are presented in Supplementary Table S1. In the log(SII) model (R^2^ = 0.213), serum iron retained its significant association (β = −0.0049 per µg/dL, 95% CI [−0.0067, −0.0031], *p* < 0.001), and BMI percentile also reached significance (β = 0.0023, 95% CI [0.0004, 0.0042], *p* = 0.018), which was not observed in the original-scale model. Similarly, in the log(NLR) model (R^2^ = 0.284), serum iron remained strongly significant (β = −0.0043, 95% CI [−0.0059, −0.0027], *p* < 0.001). The non-significant association of BMI percentile in the original-scale models likely reflects a combination of factors: limited residual variance in inflammatory indices attributable to BMI after micronutrient effects were accounted for, and the possibility that CBC-derived inflammatory indices capture downstream immunological biology more directly than anthropometric BMI classification alone.

In the exploratory obese-subgroup regression (Panel C; *n* = 39), zinc was the only variable that reached statistical significance (β = −11.912, 95% CI [−21.836, −1.988], *p* = 0.025). Each 10 µg/dL increase in serum zinc was associated with an estimated 119-unit decrease in SII within this subgroup; however, the overall model F-statistic was non-significant (*p* = 0.067), the sample was very small (*n* = 39), and no correction for multiple comparisons was applied—therefore this estimate is highly uncertain and should not be interpreted as evidence of a clinically meaningful effect. This non-significant overall model indicates that the collective predictors do not reliably explain SII variance at a conventional threshold, meaning the individual zinc coefficient—despite nominal significance—may reflect insufficient power and type I error rather than a true effect. This finding should be considered strictly hypothesis-generating.

## 4. Discussion

This exploratory cross-sectional study examined associations between a comprehensive micronutrient panel and systemic inflammatory indices in 410 children across a wide BMI spectrum. Two principal findings merit attention. First, children with obesity demonstrate significantly elevated NLR, PLR, SII, and MLR relative to peers without obesity, confirming measurable systemic inflammatory activation in pediatric obesity. Second—and most robustly—serum iron is significantly associated with NLR and SII in multivariable regression after adjustment for age and BMI percentile, with iron deficiency approximately 1.6-fold more prevalent among children with obesity. As a strictly exploratory secondary observation, zinc showed nominally significant inverse correlations with inflammatory indices within the subgroup with obesity; however, these findings derive from a small sample with a non-significant overall model and require prospective replication before any interpretation beyond hypothesis generation.

The elevation of systemic inflammatory indices in children with obesity confirmed in our data is consistent with a growing body of literature documenting adipose tissue as an active immunological organ [3,4]. Hypertrophied adipocytes and infiltrating macrophages secrete pro-inflammatory adipokines—including leptin, resistin, and tumor necrosis factor-alpha—that shift the systemic immune milieu toward a phenotype characterized by neutrophil activation, monocyte expansion, and relative lymphopenia, collectively reflected in higher NLR, SII, and MLR [4]. Notably, platelets play an integral role in two of the four indices examined (PLR and SII). Beyond their hemostatic function, platelets are recognized as active participants in immune and inflammatory responses: obesity-associated low-grade inflammation activates platelets via adipokine-mediated signaling—particularly through leptin and platelet-activating factor—leading to increased platelet reactivity, release of pro-inflammatory mediators (including thromboxane A2, P-selectin, and platelet factor 4), and enhanced platelet–leukocyte aggregate formation [14,15]. These mechanisms collectively contribute to the elevated PLR and SII observed in children with obesity in the present study and underscore that these composite indices capture not only the neutrophil–lymphocyte axis but also platelet-mediated inflammatory biology.

The robust and independent association of serum iron with NLR and SII is the primary confirmatory finding of this study. Iron is essential for neutrophil oxidative burst, lymphocyte proliferation, and cytokine-mediated effector responses [16]. Iron deficiency impairs these functions while simultaneously elevating circulating hepcidin—induced by IL-6 from adipose tissue macrophages—that blocks intestinal iron absorption and macrophage iron release, creating a vicious cycle of iron sequestration and immune dysregulation in children with obesity [17]. The clinical significance of this association is supported by effect size estimation: a shift across the interquartile range of serum iron corresponds to an estimated 155-unit decrease in SII, representing a meaningful proportion of the inflammatory gap between children with and without obesity. These findings are consistent with pediatric data demonstrating that iron status is inversely associated with inflammatory markers in children with obesity [18,19]. The 42.9% prevalence of low serum iron in children with obesity in our cohort is substantially higher than in children without obesity (27.1%), underscoring the potential clinical relevance of evaluating iron status in children with obesity.

In the exploratory subgroup analysis restricted to children with obesity, zinc showed the largest inverse correlations with SII and NLR among the micronutrients tested in that subgroup (r = −0.555 and −0.523, respectively) and was the only nominally significant predictor in obese-specific multivariable regression (β = −11.912, *p* = 0.025). These findings require cautious interpretation: only 39–40 observations with zinc data were available, the overall regression model did not achieve significance (*p* = 0.067), and no correction for multiple testing was applied to the subgroup analyses. Importantly, zinc showed no association with inflammatory indices in the full cohort, indicating that any putative effect is either specific to the inflammatory context of obesity or—more likely given the sample size—reflects a statistical artifact of a small sample with multiple comparisons. A plausible biological mechanism exists—zinc supports regulatory T-cell function and suppresses NF-κB-mediated inflammatory transcription [20,21], and obesity-related cytokine release may redistribute zinc via hepatic metallothionein upregulation [22,23]—but cross-sectional data from a small sample cannot distinguish biological specificity from chance. These observations provide a rationale for formally powered prospective investigation, not evidence of a clinically actionable effect.

Folate showed significant bivariate Spearman correlations with NLR, SII, PLR, and MLR across the full cohort but did not achieve significance in multivariable regression, suggesting that its bivariate association may be confounded by age, BMI, or correlated deficiencies. Folate participates in one-carbon metabolism, providing methyl groups for nucleotide synthesis in rapidly dividing immune cells [24]. Lower folate in children with obesity versus those without (9.2 vs. 10.8 ng/mL, *p* = 0.004) likely reflects dietary inadequacy typical of energy-dense, micronutrient-poor dietary patterns, but without dietary assessment data, its independent role cannot be established.

Ferritin showed positive associations with inflammatory indices, consistent with its role as an acute-phase reactant upregulated by IL-6 and other inflammatory mediators—rather than a reliable indicator of iron stores in inflammatory states [25]. This distinction is clinically important: elevated ferritin in the context of obesity may more likely reflect inflammatory activation than adequate or improved iron storage. Consequently, iron status in children with obesity should not be assessed from ferritin alone; transferrin saturation and, where available, serum hepcidin or soluble transferrin receptor provide more reliable markers of true iron deficiency in the presence of inflammation. Clinicians evaluating children with obesity should therefore interpret elevated ferritin cautiously, as it may mask concurrent true iron deficiency and should prompt rather than reassure. Vitamin D did not differ significantly between groups and showed modest, inconsistent inflammatory associations, likely reflecting the high background prevalence of vitamin D insufficiency across both groups (~37%), which limits between-group contrast.

### Limitations

Several limitations of the present study must be acknowledged. First, the cross-sectional design precludes causal inference: reverse causality cannot be excluded, as systemic inflammation—through hepcidin, metallothionein, and acute-phase protein responses—may itself alter circulating micronutrient levels independently of dietary intake, meaning the observed associations may reflect inflammatory sequestration of micronutrients rather than micronutrient deficiency driving inflammation. Second, the classification of iron deficiency relied solely on a serum iron threshold (<60 µg/dL). This threshold corresponds to the lower limit of the normal reference range used in our laboratory and is broadly consistent with pediatric reference standards and clinical guidelines (e.g., Soldin et al. [26]; Brugnara and Platt [27]; Baker and Greer, American Academy of Pediatrics [28]); however, serum iron exhibits considerable biologic variability and is markedly influenced by inflammatory states, rendering it an imperfect proxy for true iron deficiency. A complete diagnostic evaluation requires additional markers, including ferritin, transferrin saturation, and soluble transferrin receptor; the absence of these data is acknowledged as a substantive limitation. Additionally, because CRP and hepcidin were not measured—reflecting the routine clinical context in which blood samples were obtained—true (absolute) iron deficiency cannot be distinguished from functional iron deficiency driven by the acute-phase response; this distinction is particularly important in the context of obesity-related inflammation, where hepcidin-mediated iron restriction may elevate ferritin while simultaneously depleting functional iron. Third, the subgroup with obesity comprised only 56 children, of whom 39–40 had complete zinc and regression data; the non-significant overall F-statistic in the subgroup model (*p* = 0.067) confirms insufficient power for confirmatory subgroup conclusions. Fourth, no correction for multiple comparisons was applied, increasing the risk of false-positive findings, particularly among the numerous secondary Spearman correlations; BH-FDR-adjusted q-values are presented directly in Table 4 and confirm the robustness of iron and folate associations, while demonstrating that zinc is not significant across any index in the full cohort after correction. Fifth, several key confounders—dietary intake, pubertal status, socioeconomic status, and physical activity level—were absent from the dataset and could not be adjusted for; each independently influences both micronutrient concentrations and systemic inflammatory markers in children, representing a meaningful source of residual confounding that limits causal interpretation. Sixth, the present analysis combined children with overweight and normal weight into a single non-obesity reference group; this approach may obscure meaningful differences between children with overweight and those of normal weight, and future studies should consider including a distinct overweight category. Seventh, zinc data were missing in 50 of 410 patients (12%); if missingness was related to clinical or nutritional characteristics, this may have introduced selection bias. Eighth, the seasonality of blood collection was not systematically documented, which is a relevant limitation for vitamin D interpretation. Ninth, formal VIF diagnostics confirmed no meaningful multicollinearity among predictors (all VIF < 1.25); the pairwise correlation matrix is provided in Table S2. Finally, generalizability may be limited by cultural, dietary, and healthcare factors specific to this Turkish pediatric population.

These findings identify two specific research priorities. First, adequately powered prospective studies should formally test the association between zinc status and inflammatory indices in children with obesity, with pre-specified hypotheses, multiple comparison correction, and dietary assessment. Second, prospective interventional studies evaluating the effect of iron repletion on inflammatory indices in iron-deficient children with obesity would be a clinically motivated next step, given the consistent association observed here.

## 5. Conclusions

Children with obesity demonstrate significantly elevated systemic inflammatory indices. Serum iron shows the most consistent and methodologically robust associations with NLR and SII after multivariable adjustment, and iron deficiency is substantially more prevalent in children with obesity; these cross-sectional findings are consistent with a clinical rationale for evaluating iron status in children with obesity, though prospective evidence is needed before formal recommendations can be made. In an exploratory analysis restricted to children with obesity, zinc showed inverse correlations with inflammatory indices and was the only nominally significant micronutrient predictor in subgroup regression. These observations are strictly hypothesis-generating—the sample was small, the overall model non-significant, and no multiple comparison correction was applied—and no clinical recommendations regarding zinc can be drawn from this cross-sectional data. Prospective, adequately powered studies with dietary assessment and pre-specified hypotheses are needed to determine whether zinc status is genuinely associated with inflammatory burden in pediatric obesity and whether iron repletion modulates systemic inflammatory indices in iron-deficient children with obesity.

## Figures and Tables

**Table 1:** Demographic and hematological characteristics of the study population.

Characteristic	All (*n* = 410)	Obese (*n* = 56)	Non-Obese (*n* = 343)	*p*-Value
Age (years), mean ± SD	7.2 ± 3.8	8.1 ± 3.6	7.0 ± 3.8	0.064
Sex, Male/Female	205/205	28/28	177/177	1.000
BMI percentile, median (IQR)	23.3 (6.6–59.5)	98.0 (97.0–99.0)	17.9 (5.6–36.3)	<0.001 ***
WBC (×10^3^/µL), median (IQR)	7.4 (5.9–9.7)	8.1 (6.5–10.2)	7.2 (5.8–9.5)	0.042 *
Neutrophil (×10^3^/µL)	3.4 (2.2–5.1)	4.1 (2.6–5.7)	3.2 (2.2–4.9)	0.019 *
Lymphocyte (×10^3^/µL)	3.1 (2.4–4.0)	3.0 (2.3–3.8)	3.1 (2.4–4.1)	0.374
Platelet (×10^3^/µL)	355 (271–438)	382 (305–476)	348 (265–432)	0.031 *

BMI: body mass index; IQR: interquartile range; SD: standard deviation; WBC: white blood cell count. Bold *p*-values indicate statistical significance. * *p* < 0.05; *** *p* < 0.001. Comparisons made using the Mann–Whitney U test (continuous variables) and chi-square test (sex).

**Table 2:** Serum micronutrient levels by obesity status, expressed as median (IQR).

Micronutrient	All Patients (*n* = 410)	Obese (*n* = 56)	Non-Obese (*n* = 343)	*p*-Value
Iron (µg/dL)	77 (59–106)	64 (48–93)	79 (59–107)	0.003 **
Ferritin (ng/mL)	23 (15–37)	30 (19–44)	22 (14–35)	0.006 **
Folate (ng/mL)	10.7 (8.2–13.2)	9.2 (7.0–11.5)	10.8 (8.4–13.4)	0.004 **
Zinc (µg/dL)	86 (78–93)	84 (80–91)	86 (78–93)	0.734
Vitamin D (ng/mL)	23.0 (17.3–30.4)	22.6 (17.4–28.1)	23.0 (17.3–30.8)	0.685
Vitamin B12 (pg/mL)	434 (352–555)	447 (316–553)	434 (351–555)	0.754
Magnesium (mg/dL)	2.0 (1.9–2.2)	2.0 (1.9–2.1)	2.0 (2.0–2.2)	0.605
Phosphorus (mg/dL)	4.7 (4.3–5.0)	4.9 (4.6–5.2)	4.7 (4.3–5.0)	0.036 *

IQR: interquartile range. Bold *p*-values indicate statistically significant between-group differences. * *p* < 0.05; ** *p* < 0.01. Comparisons made using the Mann–Whitney U test.

**Table 3:** Systemic inflammatory indices by obesity status.

Index	All Patients	Obese (*n* = 56)	Non-Obese (*n* = 343)	*p*-Value
NLR, median (IQR)	1.07 (0.70–1.54)	1.30 (0.93–1.89)	1.03 (0.68–1.49)	0.001 **
PLR, median (IQR)	109.8 (81.8–131.7)	125.6 (94.2–139.3)	106.3 (80.6–130.7)	0.002 **
SII, median (IQR)	349.4 (219.0–506.5)	487.1 (316.0–715.9)	332.1 (212.2–489.9)	<0.001 ***
MLR, median (IQR)	0.16 (0.12–0.21)	0.19 (0.15–0.26)	0.16 (0.11–0.20)	<0.001 ***

NLR: neutrophil-to-lymphocyte ratio; PLR: platelet-to-lymphocyte ratio; SII: systemic immune-inflammation index; MLR: monocyte-to-lymphocyte ratio; IQR: interquartile range. Bold *p*-values indicate statistically significant between-group differences. ** *p* < 0.01; *** *p* < 0.001. Comparisons made using the Mann–Whitney U test.

**Table 4:** Spearman rank correlations between micronutrient levels and systemic inflammatory indices (full cohort), with Benjamini–Hochberg FDR-adjusted q-values.

Micronutrient	NLR r	*p*	SII r	*p*	MLR r	*p*	*n*	q (NLR/SII/MLR)
Iron	**−0.273**	**<0.001**	**−0.279**	**<0.001**	**−0.215**	**<0.001**	410	<0.001/<0.001/<0.001
Folate	**−0.255**	**<0.001**	**−0.207**	**<0.001**	**−0.254**	**<0.001**	398	<0.001/<0.001/<0.001
Ferritin	**+0.177**	**<0.001**	**+0.150**	**0.002**	**+0.197**	**<0.001**	410	0.001/0.005/<0.001
Vitamin D	−0.080	0.126	−0.087	0.093	**−0.187**	**<0.001**	373	0.120/0.190/0.001
Magnesium	**−0.139**	**0.005**	−0.098	0.048	−0.075	0.133	410	0.009/0.083/0.242
Zinc	−0.046	0.397	−0.030	0.558	−0.023	0.668	360	0.229/0.391/0.391
Vitamin B12	−0.069	0.615	+0.044	0.745	−0.031	0.540	410	0.486/0.087/0.343

Bold values indicate *p* < 0.01. NLR: neutrophil-to-lymphocyte ratio; SII: systemic immune-inflammation index; MLR: monocyte-to-lymphocyte ratio. q-values represent Benjamini–Hochberg false discovery rate (FDR)-adjusted *p*-values computed across all 21 micronutrient–index pairs; q < 0.05 indicates statistical significance after correction for multiple comparisons. Iron and folate associations remain significant after FDR correction; zinc associations are non-significant after correction across all indices.

**Table 5:** Spearman rank correlations between zinc and systemic inflammatory indices in the subgroup with obesity (*n* = 40). Exploratory analysis.

Index	Spearman r	*p*-Value	95% CI (r)	*n*
Zinc vs. SII	**−0.555**	<0.001 ***	[−0.73, −0.33]	40
Zinc vs. NLR	**−0.523**	0.001 ***	[−0.70, −0.29]	40
Zinc vs. PLR	**−0.388**	0.013 *	[−0.60, −0.13]	40
Zinc vs. MLR	−0.241	0.134	[−0.49, 0.05]	40

Approximate 95% CI for Spearman r calculated using Fisher’s z-transformation. * *p* < 0.05; *** *p* < 0.001. These are exploratory findings from a small sample; formal correction for multiple comparisons was not applied.

**Table 6:** Multivariable linear regression: micronutrients and systemic inflammatory indices.

Variable	β	SE	95% CI	*p*-Value
**Panel A: Dependent variable—SII (*n* = 356; R^2^ = 0.117; Adj R^2^ = 0.104; model F *p* < 0.001)**				
**Iron (µg/dL)**	**−3.268**	**0.580**	**[−4.404, −2.132]**	<0.001 ***
**Age (years)**	**+16.264**	**5.639**	**[5.211, 27.316]**	0.004 **
Zinc (µg/dL)	+1.173	1.360	[−1.493, 3.838]	0.389
Folate (ng/mL)	+2.984	4.934	[−6.688, 12.655]	0.546
BMI percentile	+0.450	0.602	[−0.730, 1.631]	0.455
**Panel B: Dependent variable—NLR (*n* = 356; R^2^ = 0.118; Adj R^2^ = 0.106; model F *p* < 0.001)**				
**Iron (µg/dL)**	**−0.009**	**0.002**	**[−0.012, −0.005]**	<0.001 ***
**Age (years)**	**+0.069**	**0.017**	**[0.036, 0.103]**	<0.001 ***
Zinc (µg/dL)	+0.005	0.004	[−0.003, 0.013]	0.198
Folate (ng/mL)	+0.020	0.015	[−0.009, 0.049]	0.183
BMI percentile	+0.001	0.002	[−0.003, 0.005]	0.615
**Panel C: Obese subgroup—SII (*n* = 39; R^2^ = 0.294; Adj R^2^ = 0.161; model F *p* = 0.067 †)**				
**Zinc (µg/dL)**	**−11.912**	**5.063**	**[−21.836, −1.988]**	0.025 *
Iron (µg/dL)	−3.381	2.279	[−7.849, 1.086]	0.148
Folate (ng/mL)	−5.368	20.439	[−45.427, 34.692]	0.795
Age (years)	+7.236	20.935	[−33.796, 48.268]	0.732
Sex (male = 1)	+44.273	124.352	[−199.456, 288.003]	0.724
BMI percentile	+15.709	46.628	[−75.683, 107.101]	0.738

β: unstandardized regression coefficient; SE: standard error; 95% CI: confidence interval. Bold rows: *p* < 0.05. Sex was excluded from full-cohort models due to exact sample balance (*n* = 205/sex). OLS: ordinary least squares. * *p* < 0.05; ** *p* < 0.01; *** *p* < 0.001. † Overall model F *p* = 0.067; Panel C results are exploratory.

## Data Availability

The data presented in this study are available upon request from the corresponding author due to privacy and ethical restrictions.

## Supplementary Materials

The following supporting information can be downloaded at https://www.mdpi.com/article/10.3390/children13060800/s1, Table S1: Multivariable linear regression with log-transformed outcomes (log-SII and log-NLR) as sensitivity analysis for residual non-normality (Full Cohort, *n* = 356); Table S2: Pairwise Pearson correlation matrix of regression predictors (full cohort, *n* = 356) with variance inflation factors.
