# Supplementary material for: Associations of Serum Zinc and Iron with Systemic Inflammatory Indices in Pediatric Obesity: An Exploratory Cross-Sectional Study
Source: Children (Basel). 2026 Jun 10;13(6):800. doi: 10.3390/children13060800 (PMC13297680; doi:10.3390/children13060800)
Supplement: Supplementary file 1 [file children-13-00800-s001.zip › children-4321778-supplementary.pdf]

**Table S1. Multivariable Linear Regression with Log-Transformed Outcomes (log-SII and log-NLR) as Sensitivity Analysis for Residual Non-Normality (Full Cohort, n = 356)**

| **Panel A: Dependent Variable — log(SII) (n = 356; $R^2$ = 0.213; Adj $R^2$ = 0.201; model p < 0.001)** | | | | |
|---|---|---|---|---|
| **Variable** | **β** | **SE** | **95% CI** | **p-Value** |
| Constant | **+5.6883 *** ** | **0.2258** | **[5.2442, 6.1325]** | **< 0.001** |
| Serum Iron (μg/dL) | **-0.0049 *** ** | **0.0009** | **[-0.0067, -0.0031]** | **< 0.001** |
| Serum Zinc (μg/dL) | +0.0016 | 0.0022 | [-0.0027, 0.0059] | 0.4575 |
| Folate (ng/mL) | -0.0048 | 0.0079 | [-0.0203, 0.0107] | 0.5409 |
| Age (years) | **+0.0511 *** ** | **0.0090** | **[0.0334, 0.0688]** | **< 0.001** |
| BMI Percentile | **+0.0023 * ** | **0.0010** | **[0.0004, 0.0042]** | **0.0183** |
| **Panel B: Dependent Variable — log(NLR) (n = 356; $R^2$ = 0.284; Adj $R^2$ = 0.274; model p < 0.001)** | | | | |
| **Variable** | **β** | **SE** | **95% CI** | **p-Value** |
| Constant | -0.1544 | 0.1974 | [-0.5426, 0.2339] | 0.4347 |
| Serum Iron (μg/dL) | **-0.0043 *** ** | **0.0008** | **[-0.0059, -0.0027]** | **< 0.001** |
| Serum Zinc (μg/dL) | +0.0007 | 0.0019 | [-0.0030, 0.0044] | 0.7181 |
| Folate (ng/mL) | -0.0048 | 0.0069 | [-0.0183, 0.0088] | 0.4891 |
| Age (years) | **+0.0627 *** ** | **0.0079** | **[0.0473, 0.0782]** | **< 0.001** |
| BMI Percentile | **+0.0019 * ** | **0.0008** | **[0.0002, 0.0035]** | **0.0272** |

β: unstandardized regression coefficient; SE: standard error; 95% CI: confidence interval; BMI: body mass index.

Bold rows indicate p < 0.05. * p < 0.05; ** p < 0.01; *** p < 0.001.

Log-transformation was applied to improve residual normality (original-scale Shapiro–Wilk p < 0.001 for both outcomes).

Sex was excluded from models due to exact sample balance (n = 205/sex). Predictors: serum iron, serum zinc, folate, age, and BMI percentile.

**Table S2. Pairwise Pearson Correlation Matrix of Regression Predictors (Full Cohort, n = 356) with Variance Inflation Factors**

| **Variable** | **Serum Iron (μg/dL)** | **Serum Zinc (μg/dL)** | **Folate (ng/mL)** | **Age (years)** | **BMI Percentile** | **VIF** |
|---|---|---|---|---|---|---|
| Serum Iron (μg/dL) | 1.000 | 0.161** | 0.086 | -0.065 | -0.126* | 1.05 |
| Serum Zinc (μg/dL) | 0.161** | 1.000 | 0.060 | 0.017 | -0.017 | 1.03 |

| Variable | Serum Iron (µg/dL) | Serum Zinc (µg/dL) | Folate (ng/mL) | Age (years) | BMI Percentile | VIF |
|---|---|---|---|---|---|---|
| Folate (ng/mL) | 0.086 | 0.060 | 1.000 | -0.367** | -0.140** | 1.17 |
| Age (years) | -0.065 | 0.017 | -0.367** | 1.000 | 0.262** | 1.22 |
| BMI Percentile | -0.126* | -0.017 | -0.140** | 0.262** | 1.000 | 1.09 |

* $p < 0.05$; ** $p < 0.01$. Pearson correlation coefficients presented; p-values are two-tailed.

All VIF values were well below the conventional threshold of 5, indicating no meaningful multicollinearity among predictors.

BMI: body mass index. Predictors are those included in the multivariable regression models (n = 356 with complete data).
